# Building partnerships towards strengthening Makerere University College of Health Sciences: a stakeholder and sustainability analysis

**DOI:** 10.1186/1472-698X-11-S1-S14

**Published:** 2011-03-09

**Authors:** Olico Okui, Elizabeth Ayebare, Rose Nabirye Chalo, George W Pariyo, Sara Groves, David H Peters

**Affiliations:** 1 Department of Health Policy, Planning and Management, School of Public Health, Makerere University College of Health Sciences P.O.Box 7072, Kampala, Uganda; 2 Department of Nursing, School of Health Sciences, Makerere University College of Health Sciences . P.O.Box 7072, Kampala, Uganda; 3 Johns Hopkins University School of Nursing, Baltimore, MD 21205, USA; 4 Johns Hopkins University Bloomberg School of Public Health, Baltimore, MD 21205, USA; 5 HQ/HWA Global Health Workforce Alliance, World Health Organization, Geneva, Switzerland

## Abstract

**Background:**

Partnerships and networking are important for an institution of higher learning like Makerere University College of Health Sciences (MakCHS) to be competitive and sustainable.

**Methods:**

A stakeholder and sustainability analysis of 25 key informant interviews was conducted among past, current and potential stakeholders of MakCHS to obtain their perspectives and contributions to sustainability of the College in its role to improve health outcomes.

**Results:**

The College has multiple internal and external stakeholders. Stakeholders from Uganda wanted the College to use its enormous academic capacity to fulfil its vision, take initiative, and be innovative in conducting more research and training relevant to the country’s health needs. Many stakeholders felt that the initiative for collaboration currently came more from the stakeholders than the College. External stakeholders felt that MakCHS was insufficiently marketing itself and not directly engaging the private sector or Parliament. Stakeholders also identified the opportunity for MakCHS to embrace information technology in research, learning and training, and many also wanted MakCHS to start leadership and management training programmes in health systems. The need for MakCHS to be more vigorous in training to enhance professionalism and ethical conduct was also identified.

**Discussion:**

As a constituent of a public university, MakCHS has relied on public funding, which has been inadequate to fulfill its mission. Broader networking, marketing to mobilize resources, and providing strong leadership and management support to inspire confidence among its current and potential stakeholders will be essential to MakCHS’ further growth. MakCHS’ relevance is hinged on generating research knowledge for solving the country’s contemporary health problems and starting relevant programs and embracing technologies. It should share new knowledge widely through publications and other forms of dissemination. Whether institutional leadership is best in the hands of academicians or professional managers is a debatable matter.

**Conclusions:**

This study points towards the need for MakCHS and other African public universities to build a broad network of partnerships to strengthen their operations, relevance, and sustainability. Conducting stakeholder and sustainability analyses are instructive toward this end, and have provided information and perspectives on how to make long-range informed choices for success.

## Background

Partnership and networking are important in helping institutions to be competitive, sustainable and survive. Through networking, African institutions of higher learning can improve their productivity, quality and capacity to use knowledge to transform African societies [1]. The European and Developing Countries Clinical Trials Partnership (EDCTP) has shown that networking between Europe and Sub Saharan Africa has supported African research, capacity building, advocacy, fund raising, management, and information management. This EDCTP networking has successfully created graduate study programs in Sub Saharan Africa, transfer of technology, hands on research training in the field, expanded network partnerships, and continued scientific exchange [2]. In another partnership between Denmark and Zimbabwe, partners also demonstrated that North-South networks and partnerships helped to support health sector reform and research capacity building. This partnership pointed out that in the strengthening of the African institution every effort needed to be made to integrate the African country National Health Strategic Plan with the partnership activities to have the biggest impact [3]. Both partnerships see these networks developing mechanisms for health scientists in Africa to be able to compete in a transparent and equitable basis for international funding [2,3].

Makerere University College of Health Science (MakCHS), a successor institution of the Makerere University Faculty of Medicine and the School of Public Health, the oldest and utmost public institution in Uganda for research and training of health professionals, cannot be an exception. As a result of its recent organizational transformation into the four schools of Biomedical Sciences, Health Sciences, Public Health and Medicine, MakCHS undertook to identify how the College could more effectively impact the health sector in Uganda and internationally. Its capacity to address contemporary health challenges in the country and the region on a sustainable basis is of strategic importance. Building networks and partnerships with stakeholders is, therefore, not only a pre-requisite to attaining MakCHS goals, but also to remain relevant and sustainable.

Stakeholders are individuals, groups, or organizations that have a substantive interest, role, power, or rights in the affairs of an organization [4]. Stakeholder analysis is one approach for generating knowledge about the actors so as to understand their behavior, intentions, inter-relationships and interests; and for assessing the influence and resources they bring to bear on decision making and implementation of the activities of an organization [5]. On the other hand, sustainability analysis considers the long-term ability of an organization to mobilize and allocate sufficient resources for activities that produce benefits valued sufficiently by its stakeholders [6]. Both analyses were carried out for MakCHS because they are inter-related.

This paper identifies the past, current and potential stakeholders of MakCHS, their status in relation to the College, their perspectives on the College, and their contribution to the appropriate functioning and sustainability of the College in its mission to improve health outcomes in the country and the region. The findings are relevant to MakCHS and other higher institutions of learning yearning to broaden their support base rather than largely depending on public funding.

## Methods

As part of MakCHS strategic planning process, a stakeholder and sustainability analysis was undertaken in 2009. This analysis was done as part of MakCHS and Johns Hopkins University (JHU) Collaborative Learning Initiative grant [7]. The perspectives of past, current and potential stakeholders were sought concerning the role and capacity of the College to mobilize, allocate, and utilize resources to sustainably execute its functions. In this regard MakCHS stakeholders were assessed for interests, powers, influence, as well as, involvement with the College, and the mechanisms through which they were or could be involved. Also identified were the tangible and intangible resources stakeholders deployed or were willing to deploy to prop up the College’s activities.

The study population included past, current and potential stakeholders of MakCHS, both internal and external. A cross sectional qualitative design was used. The study team conducted Key Informant (KI) interviews with stakeholder representatives. Internal stakeholders included leaders of the College, namely the principal and deans of the schools. External stakeholders included both local and international institutions and organizations from the public, non-governmental organizations (NGOs), and the private sector organizations with an interest in the College. They were also identified if they were involved with the college based on mandates and functions, were already collaborating with the College or were seen as important potential partners.

The external stakeholders were purposively sampled. The study group first listed all possible relevant external stakeholder organizations in each of the above groupings. The number was extensive and the study group then selected those organizations most interested in health care, health research and education of health professionals. Specific weight was placed on the health professions of medicine, public health, nursing and pharmacy since these are the educational programs at MakCHS. Internal stakeholders were also consulted to make sure all pertinent organizations were listed. From each of the 28 selected organizations the senior person in Uganda for the organization was identified as the Key Informant. On some occasions when the senior person was contacted they suggested another person in the organization who would have more knowledge about their relationship to Makerere University or to the health sector. That person then was identified as the KI for that organization. Of the 28 identified 25 KI were interviewed.

The specific external stakeholders interviewed comprised the Ministries of Health, Education, and Finance; Local Government, and the chairperson of the Parliamentary Committee on Social Services. Another group was the leaders of the five Ugandan Statutory Bodies related to health. Also interviewed were representatives of four faith-based organizations that have a stake in the country’s health system. Additional external key informants interviewed included representatives of a private hospital, that of the Uganda Health Consumers Organization, two multi-lateral agencies, three bilateral agencies, two international organizations and one international NGO.

The KI interviews examined the structures and mechanisms for the organization involvement, their roles, power, interest, influence, and the resources they had brought or would bring into the College or its units (schools or departments), as well as their willingness to be involved with the College. The informants were also asked to give their views on the present functioning, capacity and sustainability of the College to address contemporary health challenges in the country. They were also invited to identify other potential stakeholders with whom the College could network.

KI interview guides formulated in English were used for the interviews. The interviews were conducted in English and tape recorded with prior permission of the respondents. Hand written notes were also taken during the interviews by the interviewers themselves.

Members of the study team also reviewed seven documents (statutes) namely: *The Universities and Other Tertiary Institutions Act, 2001; Statutory Instrument Number 22, 2008 under The Universities and Other Tertiary Institutions Act, 2001 (Establishment of the College of Health Science, Makerere University) Order, 2008; Statute for Constituent Colleges of Makerere University, 2006; The Medical and Dental Practitioners Statute, 1996; Nurses and Midwives Act, 1996; Allied Health Professionals Act1996; and Pharmacy and Drugs Act 1971.* This was to gain insight into the power, influence and other relationships some stakeholders have in the College or any of its units. The structures and mechanisms for exercising such authority and influence were obtained from these documents. A check list guided the document review.

### Analysis methods

The KI interviews were transcribed and typed verbatim, and so were the hand written notes. The transcriptions were then read thoroughly by the study team. The interview transcripts and notes were compared to corroborate content and validate accuracy. Derived from the definition of stakeholders the following themes were considered for analysis: structure for stakeholder involvement, interest or power in the college, role of stakeholders in the College, the source of initiation of the relationship, and the perspective of stakeholder on the functioning and capacity of the College. Thematic content analysis was conducted by summarizing the transcriptions into categories according to meaning and frequency of occurrence. Thereafter the categories were matched with the prior identified themes in accordance with the objectives using a text matrix to facilitate analysis. Data from the document review was summarized manually according to recurring themes of influence, interest, mechanisms and contribution of the various stakeholders. The level of influence was determined by the individual respondent in their description of the relationship with MakCHS. Some had a daily influence of running the College while others were more statutory.

### Ethical approval

The Institutional Review Boards of MakCHS School of Public Health and Johns Hopkins University School of Public Health determined that the assessment did not involve human subject research considering that the views expressed by the respondents were not personal but involved providing information given about in their capacity as leaders of their organizations.

## Results

Altogether 25 key informants, representing both the College and external stakeholder institutions and organizations, were interviewed. They were senior leaders at various levels of their organizations or institutions. Table 1 provides a summary of external stakeholder characteristics and perspectives found in the KI interviews.

**Table 1:** Stakeholder characteristics and perspectives

Stakeholder	Level of Influence	Type of Influence	Support to College	Interest, Concerns, Expectations
Government Ministries/Parliament	High, much of it statutory	Policy makers & resource allocation, Statutory Power, product consumers	Approves	Want College to do research to inform policy, increase numbers & diversity of health cadres, high ethical behavior of graduates, publicity of their good work
Statutory Bodies	High, but not control on a daily basis	Accreditation of graduates, employers of graduates, influence by law	Strongly approve	Want quality training and professional conduct of graduates, sees a need for visionary college leadership, need for training in management, decentralize college to regions
Faith-Based Health Provider Organizations	Medium	Consumer of College graduates, are placement sites for training	Strongly approves	Educate students to meet needs of Ugandan people, research, have high standards for leadership and management in the College, improve college financial situation through collaborating with private sector to mobilize resources and not relying so heavily on government funding,
International organizations/NGOs	Medium	Donor/Funder and advisor to policy makers	Strongly approve	Emphasize innovative training, show strong financial management, be pro-active and show initiative to network, market itself and do research addressing priority health problems
Multi-lateral development agencies	High	Donors and advisors for capacity building and research	Strongly approve	More initiative to collaborate including with parliament, higher professional & ethical conduct of graduates and Decentralize College to other parts of country
Bi-lateral development agencies	High	Donors and advisors for capacity building and research	Strongly approve	College to take initiative to have more relevant, funded and quality research and publication. Start health management training. College needs to sell itself so that development partners know where to come in and support. More regional networking
Local Agencies	Low	Consumers of college graduates, major beneficiary of their services	Approve	Regular curriculum review to match the needs of the constituency, better public relations and marketing functions for College, strengthen research capacity by bringing in other partners and increase publications. College leadership to be bold and daring! Engage private sector more

### Stakeholders, current and potential

A number of stakeholders or potential collaborators of the College or its constituent units of varied categories were identified or mentioned by the KIs. They included:

(a) Government Ministries of Health (MOH), Education & Sports (MOES), Finance (MOF) and Local Government( MOLG); District Local Governments, and Parliament, in particular the Social Services Committee;

(b) Statutory Bodies: Uganda Medical and Dental Practitioners Council (UMDPC), Uganda Nurses and Midwives Council (UNMWC), the Health Service Commission (HSC), the Pharmacy Board, and the Allied Health Workers Council, Health Service Commission (HSC), and Uganda Virus Research Institute;

(c) Faith-Based Organizations: Uganda Catholic Medical Bureau, Uganda Protestant Medical Bureau, Uganda Muslim Medical Bureau, and the Joint Medical Stores;

(d) International organizations and NGO: International College for Health Cooperation in Developing Countries, German Academic Exchange Service, Bill and Melinda Gates Foundation, Rockefeller Foundation;

(e) Multi-lateral agencies: United Nations Fund for Population Activities, United Nations Children's Emergency Fund (UNICEF) and World Health Organization;

(f) Bi-lateral Agencies: United Kingdom Department of International Development, Danish International Development Agency, European Union, Canadian International Research Center, Swedish International Development Agency, United States Agency for International Development, Center for Disease Control and Prevention, Italian Cooperation;

(g) Local Agencies: private hospitals, Uganda National Health Consumers Organization (UNHCO);

(h) other universities, both local and foreign: Gulu University, Mbarara University, Johns Hopkins, Columbia, Alberta, Muhimbili, Moi, and MacMaster.

Notable in their absence were the local private business sector. All of our repeated efforts to obtain interviews with them were unsuccessful.

### Stakeholder power

The Ministry of Education & Sports (MOES) is directly responsible for the University and exercises power over the College through *The Universities and Other Tertiary Institutions Act, 2001*. This Act sets up The National Council for Higher Education to implement its provisions. The Act empowers the NCHE to among other things ensure minimum standards for courses of study and to establish any university unit(s) as a constituent college of a public university. Indeed it is under this Act that the MakCHS was established as a constituent college of Makerere University in 2008.

Some of the statutory bodies also have some powers over the activities of the College or its individual departments. The UMDPC, UNMWC and Pharmacy Board are mandated by law to regulate medical, nursing and pharmacy training standards, respectively. They have power to withhold recognition or accreditation of the respective units of the College under the law (*The Medical and Dental Practitioners Statute, The Nurses Midwives Act, 1996 and Pharmacy and Drugs Act, 1971*). The College is represented on these bodies. So far none of these bodies have found it necessary to evoke the use of their powers with respect to College activities, but these powers do give the bodies some influence and leverage over the College.

The UMDPC, UNMWC and Pharmacy Board also have power to control medical, nursing and pharmacy practice, as well as, promote professional and ethical practices of these professionals in the country. Some key informants including some from these bodies alluded to the need for more vigorous training to enhance professionalism and ethical conduct among College graduates. This quote summarizes their views.

*“Professionalism of College graduates is wanting; training is lacking something ethically. Health workers of these days are not committed to their work. They lack ethics in the way they do their work.”* (KI, statutory body).

### Stakeholder influence

The stakeholders that have some power by law over MakCHS (e.g. MOES, UMDPC) exercise influence through legal means. However, most stakeholders that were mentioned, in particular international organizations and foreign collaborating universities, exercise influence through funding research activities and staff and infrastructure development, thus having leverage over decisions about the research activities in the College and about staff as well as infrastructure development.

### Stakeholder interests in, perspectives about the capacity and functioning of the college

Some stakeholders reported that they fund College activities because their interest is to develop the capacity of the College to address what they perceive to be the country’s priority health needs. The following quote captures the stakeholders’ view about the college’s capacity and in what direction they would wish it to be channeled:

*“The College has very good academic capacity with a concentration of talent. We want technical support from the College to generate evidence through operational research to intervene in priority problems.”* (KI, donor agency).

However, a KI from the College leadership put it differently:

*“These institutions and organizations have missions; therefore what motivates them to collaborate with us is what we do that enhances what they do.”* (KI, MakCHS leadership)

These apparently divergent views reflect different interpretations of the interests of the stakeholders in the College by the stakeholders themselves and the college management. However, both internal and external stakeholders interviewed were more or less unanimous that the initiative for collaboration was coming more from external collaborators rather than the College. This makes research activities at the College donor driven. An internal stakeholder in admitting this said:

*“As the College, we do not sell ourselves to the outside world. The College needs to become very innovative, creative and strategic and seek information in knowing the player out there.”* (KI, MakCHS leader)**.**

International and bilateral organizations concurred:

*“The College needs to sell itself.”* (KI, NGO)

Some of the stakeholders have an investment in the College’s activities because they are consumers of its products, namely the graduates that offer services in the health care system. In this category are HSC, the faith-based NGOs, districts and the private hospitals. It was even suggested that some of their facilities could be used for training purposes. Indeed districts provide accommodation to the students free of charge while they are doing field study/work. The clients/patients interests were reportedly represented by Uganda National Health Consumer Organization with a view to protecting and promoting patients’ rights.

### Structures and mechanisms for stakeholder involvement

The mechanisms and structures through which the stakeholders collaborate with the College were reported to be either contracts or memoranda of understanding. The line ministry and the statutory bodies have national laws defining structures for relating with the College while a member of UNHCO sits on the Institutional Review Boards of the Schools of Medicine and Public Health. However, the College is in the process of putting in place the needed structures and mechanisms for mobilizing and managing grants.

*“We need a development office, grant and contracts office to fund raise, allocate and manage the funds,”* (KI, MakCHS leadership)

Table 1 provides a summary of external stakeholder characteristics and perspectives found in the KI interviews. The stakeholders’ perspectives about the college were reflected as interests and as concerns. They were also expressed as expectations so as to enhance the proper functioning of the college in order to ensure sustainability. All these included among others the need for the college to put more emphasis on research, training in ethics, training in management and networking more widely as well as engaging with the private sector.

### Stakeholder power mapping

**Figure 1:**
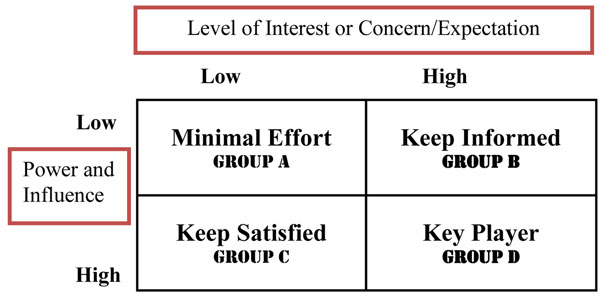
Critical stakeholder mapping for the college. [9,10]

Figure 1 maps the current stakeholders on the basis of their level of power, influence and interest in the functioning and development of the college vis-a-viz their role and support given to the college. Stakeholders were categorized on a perceived rating of level of power and influence against their role, interest, as well as, their expectations on the development and functioning of the college as a first rate institution The stakeholders were broadly grouped into 4: (A) low interest/low power, (B) high interest/low power, (C) low interest/high power, and (D) high interest/high power.

• Group A requires the College to focus minimal effort. They, in general, do not pose a serious threat to the College. The group would include private health care providers and the private business sector.

• Group B, despite their high interest, have little direct influence over the College, and simply need to be kept informed about college business. These may include HSC, faith-based organizations and civil society organizations.

• Group C, may or may not realize the degree of effect they have over the College but definitely must be kept satisfied. Withdrawal of their support can be catastrophic to College development and functioning. These include some government ministries, parliament and civil society.

• Group D are the most important constituency for the college and must be kept satisfied because they have interest, power, and influence over the college. In this group one would put MOES, MOH, statutory bodies in health and the donors.

### Sustainability

For this assessment we adopted Olsen’s definition of sustainability, “An institution is sustainable when operated by a system with long-term ability to mobilize and allocate sufficient and appropriate resources (manpower, technology, information and finance) for activities that meet public needs/demands” [8]. He emphasized that an organization’s ability *to produce certain desired activities and support functions ( benefits)* should be sustained and be linked to the position of its stakeholders, internal and external. Therefore the ability of MakCHS to ensure adequate resources for its activities is closely linked to its capacity and activity profile, as well as, the role and contributions of its present and future stakeholders, the context notwithstanding.

*“The College is full with capacity to use resources and train good health professionals because it has good brains behind it, for example it has a good number of professors and other good professionals.”* (KI, International NGO).

However, the College is operating in a resource constrained environment and is under-funded. It is relatively ‘young’ and is in the process of putting in place structures to acquire the needed organizational capacity to execute its mandate effectively and efficiently. There was a view expressed by some study participants that since the College is in its ‘infancy’, it needs leaders, managers, and planners who can think big to move it forward, not just academicians. In this regard KIs suggested that the college network widely, taking initiative to market itself in order to mobilize and use resources effectively, including resources from the private sector to help the College do its work.

*“It [MakCHS] has to come up with a strategic plan which will enable it to profitably and effectively utilize resources. Funds should be directed towards useful activities like improving infrastructure and capacity in training and research among others.”* (KI, international NGO)

*“Having a vision; the College should not think small. Let it spread outside Kampala. For example, acquire land for expansion in areas like Mbale, Fortportal, Kabale, Arua, so that all regions in the country can easily be served by the College.”* (KI, multi-lateral agency)

Another view was echoed for the College to develop into a research institution to generate new knowledge relevant to solving the health needs of the country and share that knowledge widely. A suggestion to embrace new technologies was also made.

*“Furthermore, they have to show that they are not only a learning institution, but a place where new knowledge can be generated. It should therefore engage in relevant research which can change the existing events for the better. It should pronounce itself as an institution which imparts extra-ordinary skills and a place with necessary admirable attributes.”* (KI, international health agencies)

*“Finally, resources should be directed towards quality training and research, modern technological advancement be embraced and encourage the collaboration with the private sector.”* (KI, local health NGO)

Most external KIs expressed the view that the initiative should come from the College and it should be relevant.

*“The College should start the initiative.”* (KI, multi-lateral agency)

A leading internal stakeholder from within the College was in agreement with most of the views expressed by other stakeholders thus:

“As the College we need to become very innovative, creative and strategic. We must do better than others; we need to fight for quality assurance, we need more land for expansion, we need more funds to upgrade our facilities. The College needs more funds to carry out research that is meaningful and relevant to this country and be able to translate this research into practice.

*Mobilizing resources is a big challenge we need to put formal structures for managing resources; and reporting to the funders is also a challenge. Allocation becomes difficult when you have a challenge of mobilizing and extremely less resources. We need a development office, grant and contracts office to fund-raise, allocate and manage the funds. We have it in plan that this office will do this task on a day-to-day basis. We need to embrace ICT whether it is in research, management, education because it will enable us to tap more opportunities elsewhere.”* (KI, MakCHS leader)

## Discussion

In this section we discuss some of the implications of the findings of this study towards building the needed partnerships to strengthen not only MakCHS but other African public universities and contribute to their sustainability. It would seem instructive for African public universities first and foremost to conduct stakeholder and sustainability analyses of their own. This will reflect their own history, present situation and performance in relation to the priority needs of their country and the world. It will provide them with information and perspectives of how to proactively forge a way forward to success.

Makerere University, the parent institution of the College of Health Science, is a public university. Core funding for the University comes from the Ugandan tax payer, which has been the case for so long that a laissez-faire attitude seems to have taken hold on its staff and management. However, the prevailing economic realities in the country make it mandatory for the University to take the initiative to network widely, as well as market itself in order to mobilize the needed resources to do its work and start new relevant programs and embrace new technologies.

Several participants pointed to the need for the College to develop into an institution that emphasizes conducting research to generate new knowledge relevant to solving the health needs of the country. It should share that knowledge widely through publications and other forms of dissemination; network with partner institutions locally, in the region, and internationally. It would seem from this view that the College should be aiming at becoming more of a research institution. Given the disease profile in the country, this may be rather premature.

The College ought to take initiative to engage the private sector more in order to tap non-traditional sources of financial support through adopting new management ideas and technologies. In order to do this it needs strong leadership and management support structures to inspire more confidence among its stakeholders, present and potential. The structures are mostly in their formative stages. However in widening its network of collaborators, it should be clear about its strategic interests, areas of focus and priorities as well as having clear ideas about the choice of stakeholders that will yield for it greater returns. In this respect a fairly long-term strategic and a business plans adopted after wide consultation would be an advantage.

The view expressed by some study participants about non-academicians leading and managing the College may be debatable for several reasons. Makerere University Medical School that is the forerunner to the MakCHS has had a history of academic excellence under the leadership/management of professional health academicians. Furthermore, it can be credibly argued that the College is a knowledge-based institution that is too specialized to be entirely led and managed by non-health professional academicians. Rather what is needed is the setting up of functional support management structures staffed with professional managers. This issue may need further examination drawing experience from other institutions in similar contexts.

## Conclusions

MakCHS has several past and present stakeholders that have supported research, staff and infrastructure development. It has therefore maintained its reputation as a leading institution, albeit ceding some initiative in the process. However, MakCHS has now the opportunity to re-capture the imagination of society in general and its current and would-be stakeholders in particular. It needs to develop a long-term vision to proactively initiate and conduct relevant research and market itself locally, regionally and internationally through publications and other means of communication. Furthermore, it ought to start new relevant programmes and embrace new technologies and methodologies to strengthen its research and training capacity. In order to do all these as well as attract more collaborators, initiative, networking widely including engaging local stakeholders more and building stakeholder confidence in its management systems will be essential in mobilizing the needed resources.

## List of abbreviations used

EDCTP: European and Developing Countries Clinical Trials Partnership; HSC: Health Service Commission; JHU: Johns Hopkins University; KI: Key informant; MakCHS: Makerere University College of Health Sciences; MOES: Ministry of Education and Sports; MOF: Ministry of Finance; MOH: Ministry of Health; MOLG: Ministry of Local Government; NCHE: National Council for Higher Education; NGO: Non-governmental organization; UMDPC: Uganda Medical and Dental Practitioners Council; UNHCO: Uganda National Health Consumer Organization; UNMWC: Nurses and Midwives Council.

## Competing interests

The authors declare that they have no competing interests.

## Authors' contributions

OO participated in the conception and design of the study, participated in the data analysis, and drafted the manuscript. EA participated in the conception and design of the study and the data analysis and helped draft part of the manuscript. RC participated in the conception and design of the study and the data analysis. SG participated in the draft of the manuscript. GP and DP participated in the conception and design of the study and helped in the drafting of the manuscript. All authors read and approved the final manuscript.
